# Hollow-core fiber gas lasers [Invited]

**DOI:** 10.1038/s41377-026-02256-y

**Published:** 2026-04-20

**Authors:** Zefeng Wang, Wenxi Pei, Zhiyue Zhou, Xuanxi Li, Hao Li, Binyu Rao, Linyong Yang, Rong Zhao, Chenxin Gao, Xiaoming Xi, Jing Shi, Guorui Lv, Luohao Lei, Qi Chen, Tianyu Li, Zhixian Li, Meng Wang, Zilun Chen

**Affiliations:** 1 College of Advanced Interdisciplinary Studies, National University of Defense Technology, Changsha, 410073 China; 2 Nanhu Laser Laboratory, National University of Defense Technology, Changsha, 410073 China; 3 Hunan Provincial Key Laboratory of High Energy Laser Technology, National University of Defense Technology, Changsha, 410073 China

**Keywords:** Fibre lasers, Solid-state lasers

## Abstract

Recent years have witnessed significant advancements in hollow-core fiber gas lasers (HCFGLs), driven by developments in hollow-core fiber (HCF) design and fabrication. These novel lasers are characterized by hollow-core structure, providing an ultralong and tiny interaction region for light and gases, thereby enabling light amplification with high efficiency, high beam quality, and tunability. HCFGLs has achieved broadband emission spanning from ultraviolet to mid-infrared (MIR) region, with a record output wavelength exceeding 4.8 μm in silica-based fiber lasers. Output power has surpassed 100 W in the near-infrared and reached an impressive 21.8 W in the MIR region. With the continuing reduction of attenuation in HCFs, particularly in the MIR regime, HCFGLs holds promises for potential applications in trace gas detection, space communication, polymer processing, medical treatment, national defense, etc. In this review, we focus on the basic principles and research progress of HCFGLs. The paradigm shifts history of HCFs are reviewed first. The development history and representative works of HCFGL based on the population inversion and stimulated Raman scattering are then introduced in detail. Finally, the future trends toward power enhancement, spectral expansion, and practical applications are also outlined. We hope this review will provide valuable insights for HCFGL researchers and interested readers, while also offering potential pathways to achieve otherwise challenging laser wavelength outputs and further power scaling.

## Introduction

The pursuit of advanced laser sources with capabilities that extend wavelength and increase power has been a driving force in photonics research, particularly due to the wide range of applications in industrial processing, medical procedures, communication systems, and military defense^1,2^. Hollow-core fiber gas lasers (HCFGLs) have emerged as a compelling solution and an innovative class of light sources with the advent of hollow-core fibers (HCFs)^3–6^. HCFGLs utilize the gas within the HCF as the gain medium, while the HCF serves as the waveguide structure that confines the light and the gas. This collaboration perfectly merges the compact structure, high conversion efficiency, and excellent beam quality of fiber lasers with the flexible wavelength selection, narrow linewidth, high damage threshold, and weak nonlinear effects characteristic of gas lasers^7,8^. This integration offers a novel approach to addressing the technical challenges that traditional solid-core fiber lasers face in terms of power scaling, wavelength expansion, and spectral line control. In particular, it holds significant potential for generating mid- to far-infrared laser bands, which are crucial for various applications but difficult to achieve with conventional fiber lasers^7,9^.

Compared to traditional gas lasers that employ large-volume, short-interaction-length gas cavities, HCFs provide an ideal environment for light-gas interaction^4^. The core region of HCFs, which can be tens of micrometers in diameter and an ultra-long length even up to kilometer-level, allows for more extensive and effective interaction between light and gas. The host material commonly used for HCFs is technologically mature silica, which offers the stability required for long-term operation, in contrast to other mid-infrared (MIR) fiber materials like fluorides and chalcogenides with poor chemical stability^10^. Moreover, HCFs have the potential for higher power output compared to these fibers, as the light field propagates mainly in the core region with minimal overlap with the cladding material, significantly overcoming loss limitations caused by material absorption^11–13^. The intensity of the light field in the overlapping region with the cladding material is at least an order of magnitude lower than that at the core center, greatly enhancing the damage threshold of HCFs. They also exhibit characteristics of low latency, low dispersion, and weak nonlinear effects, which have garnered widespread attention globally^14–16^.

Based on their working principles, the HCFGLs are primarily divided into two categories: one based on the intrinsic absorption transitions between the vibrational-rotational energy levels of gas molecules^17^, and the other one based on nonlinear effects such as the stimulated Raman scattering (SRS) of gas molecules^4^. Most common gases have vibrational-rotational energy level absorption wavelengths between 1 and 2 μm, while their transition wavelengths are generally in the MIR range^7^. Therefore, using 1 and 2 μm fiber lasers as pump sources can relatively easily achieve effective MIR laser output. HCFGLs based on this mechanism demonstrate a low threshold, which enables easy achievement of continuous-wave (CW) operation, although requiring stringent precision in pump linewidth control and wavelength stabilization. Due to the large Raman frequency shift in gases, the HCFGLs based on SRS are capable of generating MIR laser emissions by leveraging the well-established 1.5 μm fiber lasers as their pump sources^18,19^. While exhibiting significantly greater flexibility in pump wavelength selection, SRS-based HCFGLs universally require higher threshold powers, especially when operating in the MIR range. To elucidate the fundamental differences between these two lasers, Table 1 systematically compares their critical parameters, revealing their complementary advantages.

**Table 1:** Characteristics comparison of two HCFGLs

Parameters	HCFGL based on population inversion	HCFGLs based on stimulated Raman scattering
Gain mechanism	Intrinsic absorption transition	Nonlinear effects
Demand for pump source	Precise absorption wavelength Wavelength fluctuation <±1 pm (typ.^a^) Linewidth: hundreds of MHz	No wavelength selectivity Linewidth: ~GHz
HCF core diameter	Large core for reducing Mid-infrared loss (typ. **>**50 μm)	Small core is preferred (for reducing SRS threshold)
Typical gases	C_2_H_2_, HBr, CO_2_, CO, etc.	H_2_, CH_4_, D_2_, etc.
Output laser wavelength	Usually mid-infrared	Ultraviolet to mid-infrared
Threshold	Low (typ. hundreds of milliwatts)	High (typ. hundred W to kW)
CW/Pulse compatibility	Both achievable Mostly operates in CW mode	Pulse dominates due to its high thresholds
Beam quality	Excellent (typ. *M*^*2*^ < 1.2)	Excellent (typ. *M*^*2*^ < 1.2)

^a^Typical value

The development of HCFGLs is closely linked to the evolution of HCFs. In 1999, the photonic bandgap HCF (PBG-HCF) was first demonstrated^3^. Subsequently, the first experiment on hydrogen SRS inside HCFs was conducted, marking the beginning of a new era in leveraging HCF to achieve laser-gas interactions^4^. The transmission bands of early PBG-HCFs were primarily limited to the visible and near-infrared (NIR) spectral regions with relatively narrow bandwidths. However, since the vibrational-rotational energy level transitions of gas molecules typically correspond to MIR wavelengths, initial research efforts were mainly directed towards HCFGL utilizing Raman scattering mechanisms. Since 2010, with the development of anti-resonant HCFs (AR-HCFs) that have low transmission loss in the MIR band^20–22^, MIR HCFGLs based on the intrinsic absorption transitions of gas molecules have rapidly advanced. To date, with the continuing reduction of attenuation in the MIR regime inside HCFs, gases reported include C_2_H_2_, CO_2_, CO, HBr, N_2_O, and others, with output wavelengths covering various segments of the 3–5 μm range^7,17,23–26^. Simultaneously, HCFGL based on SRS has also been extended to the MIR band.

This review aims to provide an overview of the current state and prospects of HCFGLs. We hope the review can serve as a general introduction to the progress of HCFGLs and outline some interesting problems to work on soon. The review is organized as follows. We first introduce the two primary types of HCFs and their operating principles, development history, and the latest advancements. Then the next two sections cover the basic principles of HCFGLs and their respective features. The latest research progress in HCFGLs, including laser output at different wavelength bands, experimental techniques, and theoretical models, is also given. The challenges faced by HCFGLs and potential future directions for the field are discussed in Section “Challenges and Future Prospects”. Section “Conclusion” is devoted to the outlook and conclusion of the review.

## Overview of HCF

HCF represents a specialized optical fiber structure characterized by a hollow core surrounded by glass cladding. Since its successful demonstration in 1999^3^, this innovative fiber technology has significantly advanced modern optical science due to its unique properties and practical applications. HCF serves as an exceptional transmission medium and experimental platform across multiple scientific domains, including laser technology, nonlinear optics, sensing applications, communication systems, quantum mechanics, and biological research^27–31^. Based on their transmission mechanisms, HCFs can be primarily categorized into two distinct types: PBG-HCF and AR-HCF, despite their varying structural configurations.

### PBG-HCF

In 1991, Russell pioneered a novel hollow-core waveguide concept that employed a two-dimensional photonic bandgap to address the limitations of material attenuation, dispersion, and nonlinear effects inherent in conventional solid-core fibers^32^. Building upon this foundation, Russell’s research team developed advanced fabrication techniques and successfully demonstrated the first solid-core photonic crystal fiber (PCF) in 1996^33^. The subsequent breakthrough came in 1999 with the realization of the first genuine HCF, i.e., PBG-HCF^3^. This innovative fiber structure features a cladding with a periodic arrangement of air holes surrounding the core, creating a two-dimensional photonic bandgap effect. This unique configuration confines light transmission within the core by reflecting wavelengths that fall within the bandgap range, effectively preventing their propagation through the periodic air holes.

Despite the initial limitation of fiber lengths to mere tens of centimeters, the remarkable potential demonstrated by HCF technology attracted significant scientific attention. This potential was further substantiated by Corning, who achieved a low-loss PBG-HCF with a transmission window exhibiting 30 dB/km loss across a 125 nm wavelength range, reaching a minimum loss of 13 dB/km at 1500 nm^34^. Subsequent advancements by the University of Bath pushed the boundaries further, reducing the transmission loss to 1.72 dB/km at 1565 nm^35^. However, further reduction of transmission loss has proven challenging, primarily due to the scattering effects caused by surface roughness at the core boundary^36^.

Subsequent research on PBG-HCFs has primarily focused on addressing these technical challenges, with particular emphasis on maintaining low loss while expanding the transmission bandwidth. A PBG-HCF with a bandwidth of 458 nm and a loss of 6.5 dB/km at 1633 nm has been demonstrated^37^. PBG-HCFs demonstrate exceptional performance in the NIR spectrum, characterized by both low transmission loss and low bending loss^37–40^. The fiber’s small core diameter enables effective confinement of laser radiation within a micron-scale hollow core, which not only facilitates long-distance optical transmission but also significantly enhances light-matter interactions. This fundamental characteristic has served as the foundation for the development of HCFGLs^4^.

**Fig. 1:**
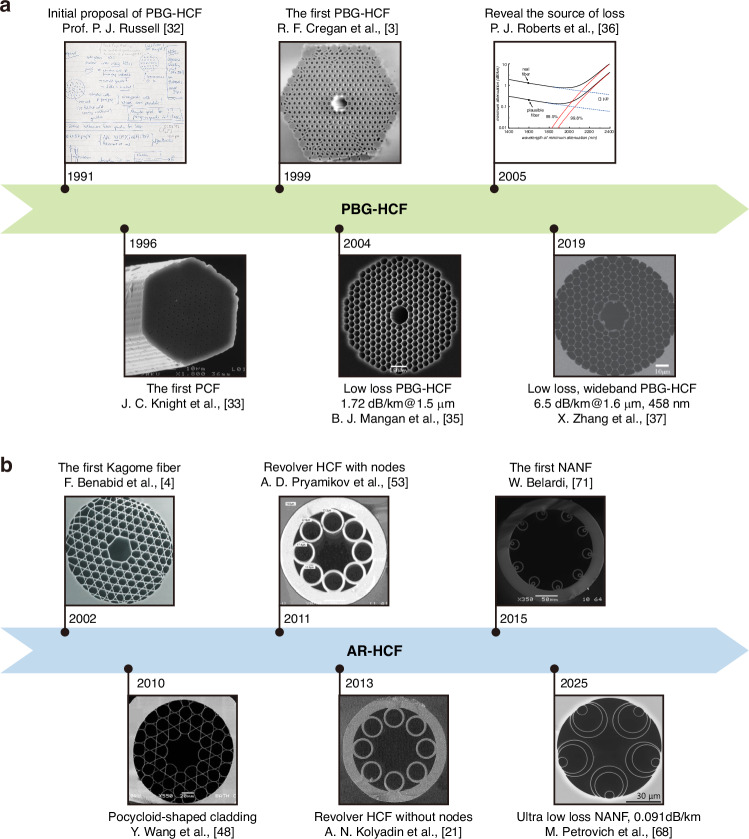
The historical progression of HCF. Identified significant milestones and HCF presentations to assess the progression of **a** Initial proposal of PBG-HCF. Reprinted with permission from ref. ^32^ © Chinese Laser Press. The first PCF. Reprinted with permission from ref. ^33^ © Optical Society of America. The first PBG-HCF. Modified from R. F. Cregan et al., Science, 10.1126/science.285.5433.1537 [1999], AAAS^3^. Low-loss PBG HCF: 1.72 dB/km @ 1.5 μm. Reprinted with permission from ref. ^35^ © Optical Society of America. Reveal the source of fiber loss. Reprinted with permission from ref. ^36^ © Optical Society of America. Low-loss, wideband PBG-HCF. Reprinted with permission from ref. ^37^ © Optical Society of America. **b** The first Kagome fiber. Modified from F. Benabid et al., Science, 10.1126/science.1076408 [2002], AAAS^4^. AR-HCF with pocycloid-shaped cladding. Adapted with permission from ref. ^48^ © Optical Society of America. Revolver HCF with nodes. Reprinted with permission from ref. ^53^ © Optical Society of America. Revolver HCF without nodes. Reprinted with permission from ref. ^21^ © Optical Society of America. The first NANF. Reprinted with permission^71^ from Institute of Electrical and Electronics Engineers. Ultra-low-loss NANF with attenuation of 0.091 dB/km. Reprinted with permission from ref. ^68^ © Springer Nature

However, the intrinsic properties of the PBG structure impose limitations on PBG-HCFs, particularly in terms of their relatively narrow transmission bandwidth. This characteristic presents challenges for transmitting non-NIR wavelengths, thereby restricting the fiber’s applicability across broader spectral ranges. Moreover, PBG-HCFs require a porous periodic structure to form a perfect PBG, hence the structure is complex and prone to high fabrication errors. These reasons have impeded the practical application process of PBG-HCF. The main historical development and technological progression of PBG-HCFs in recent years are systematically illustrated in Fig. 1a.

### AR-HCF

The breakthrough of the limitations of PBG-HCF comes from the emergence of AR-HCF. The guiding mechanism of AR-HCF fundamentally differs from that of PBG-HCF, operating on the anti-resonant reflecting optical waveguide principle^41^. In this configuration, the micro-structured thin wall forming the core boundary functions similarly to a Fabry-Perot cavity. Light satisfying the cavity’s resonance condition escapes into the thin wall, while non-resonant wavelengths experience anti-resonant reflection, effectively confining them within the core. This mechanism endows AR-HCF with multiple transmission bands, where high-loss regions correspond to resonant wavelengths and low-loss regions to anti-resonant wavelengths, with the resonance condition determined by the core boundary’s wall thickness. Figure 1b illustrates the historical development of AR-HCF technology.

The pioneering AR-HCF was the Kagome-structured fiber unexpectedly introduced by Benabid in 2002^4,42^. This fiber, unlike PBG-HCFs, features increased spacing between the cladding’s periodic air holes and provides an extraordinarily broad light transmission band. In the investigation of the guiding mechanism of Kagome AR-HCF, more emphasis was placed on the optimization of the cladding geometry without paying special attention to the core structure^43–46^. In order to illustrate the impact of the core surround, Wang introduced an innovative Kagome AR-HCF using a hypocycloid-shaped core^47,48^. The incorporation of a hypocycloid structure at the core-cladding interface markedly diminished the overlap between the core mode and cladding material. Subsequent refinements diminished the loss of the hypocycloid-shaped Kagome AR-HCF over many wavelength bands^49–52^. Ongoing research has revealed that the Kagome-structured cladding does not substantially influence the properties of the fiber. The efficacy of Kagome fibers is largely ascribed to their epitrochoid-shaped cores, rather than the Kagome-structured cladding. The design of HCFs has been streamlined, leading to the creation of AR-HCFs that consist of a singular layer of air holes. The main breakthrough lies in the revolver AR-HCF, which arranged a single ring of touching capillaries around a hollow core^53^. Many subsequent low-loss AR-HCFs were also improved based on this^20,54^. This structural transformation also significantly reduces the difficulty of HCF manufacturing, avoids a series of engineering problems, and is conducive to the maintenance of microstructure during the fabrication process.

Subsequent investigations into the transmission properties of AR-HCFs have demonstrated that contact points between adjacent hollow capillaries in the cladding may result in increased transmission losses^55^. In 2013, Kolyadin et al. developed a nodeless AR-HCF for light transmission in the 2.5–7.9 μm waveband, with a measured loss of 50 dB/km at a wavelength of 3.39 μm^21^. The experimental findings show that, in contrast to AR-HCFs with nodes, nodeless AR-HCFs not only significantly reduce the transmission loss of the fiber but also render the loss curve more uniform. This conclusion was further reinforced in 2014 when Belardi et al. conducted comparative theoretical analyses of bending loss characteristics, demonstrating that node elimination significantly suppresses bending-induced losses in HCFs^54^. Subsequently, the benefits of nodeless AR-HCFs in laser transmission, little transmission loss, and extensive transmission bandwidth have been consistently documented^56–59^.

A novel design of AR-HCF was introduced in 2014, known as the Nested Anti-Resonant Nodeless Fiber (NANF)^60^. Structural optimizations of NANFs have revealed their potential to surpass conventional performance limits, with theoretical models predicting attenuation values below 0.1 dB/km—outperforming both PBG-HCFs and solid-core fibers^60–63^. The technology reached a critical benchmark in 2019 with the first demonstration of a HCF achieving below 1 dB/km loss (0.65 dB/km)^64^, followed by progressive improvements reducing C + L band attenuation to 0.28 dB/km by 2020^65^. In 2022, the triple-nested NANF exhibited a loss of 0.174 dB/km in the C-band, which was inferior to the loss of commercial G.652 fibers^66^. In 2024, Microsoft, in partnership with the University of Bath, announced the production of a NANF exhibiting three independent loss measurements of <0.11 dB/km at 1550 nm^67^. Recently, the team further reduced the loss to 0.091 dB/km, marking the lowest attenuation yet documented in optical fibers^68^.

After nearly 30 years of development, the transmission loss of HCF has been greatly reduced and has surpassed that of solid-core silica fiber across multiple spectral bands^69,70^. In review of the history of HCF, significant improvements in optical performance were made by the introduction of a negative curvature core surround, then by fiber topologies with a single layer of non-touching tubes, and finally by the addition of small nested tubes to increase the number of coherent air/glass reflections in the radial direction and thus reduce confinement loss—a design known as NANF^65^. This distinctive characteristic makes AR-HCFs particularly attractive for MIR applications, where silica glass exhibits substantial absorption losses. Silica-based AR-HCF has shown low loss comparable to commercial fluoride fibers in the MIR band^11^ and has successfully extended the transmission band to ~8 μm^21^. NANF demonstrates lower transmission loss in the MIR band, and research has indicated that NANF possesses significant bending resistance within the same band^71,72^.

As the transmission loss of AR-HCF gradually becomes lower than that of traditional solid-core fiber, AR-HCF is particularly suitable for optical communication that requires long-distance transmission. For communications, the main development trend is to standardize the structural parameters so that HCFs from different manufacturers can be interconnected. Secondly, it is necessary to continue to reduce the transmission loss of HCFs and expand the transmission bandwidth. At the same time, it is necessary to reduce the bending loss (including macrobending loss and microbending loss) and improve the mechanical stability of optical fibers. The light-guiding range of AR-HCF can be flexibly tuned by changing the capillary wall thickness, making it the predominant fiber type in HCFGLs. However, very low losses are of only secondary importance due to the relatively short HCF lengths used. What is more important is the expansion of transmission bandwidth, which requires considering low-loss transmission in both the pump and the laser band. This is not difficult for the NIR band, but the main challenge lies in the MIR band, especially above 4 μm. This necessity has led to the exploration of soft glass materials with low loss in the MIR range.

## HCFGLs based on population inversion

**Fig. 2:**
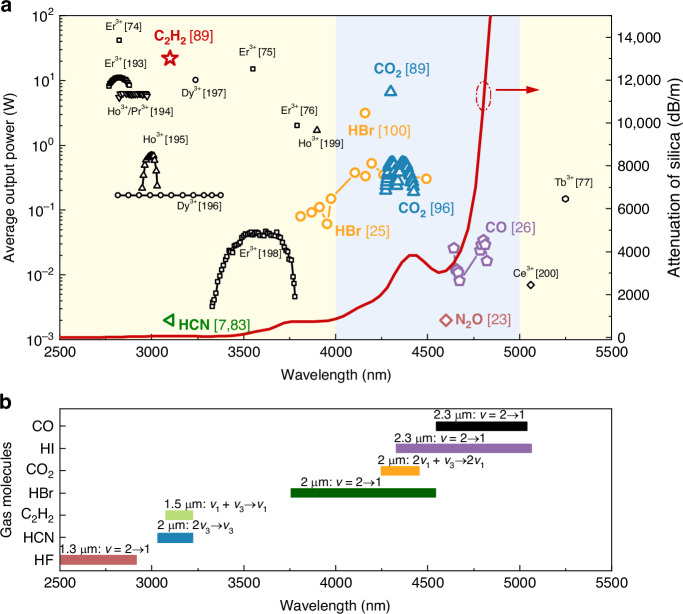
Summary of the population inversion type HCFGLs. **a** Summary of the state-of-art advancements in MIR fiber lasers regarding average output power, lasing wavelengths, and the absorption characteristics of bulk silica^7,23,25,26,74–77,89,92,96,100,193–200^. The black data indicate the performance of RE-doped fiber lasers, whereas the colorful data illustrate the advancements of HCFGLs. The absorption loss of bulk silica is depicted by the red curve (right *y*-axis) and originates from Heraeus F300 fused silica glass^11,107^. From a wavelength perspective, HCFGL predominantly resides in the 4–5 μm range, necessitating the use of two background colors to differentiate the wavelength regions. The 3 μm HCN^7,83^ and 4.6 μm N_2_O^23^ lasers are also depicted in the figure; however, they do not reflect the true average output power due to insufficient data. Given that the OPO pump sources employed by the two lasers usually exhibit reduced repetition rates, they are represented at a lower level. **b** Emission spectra of several candidate gases, including those that have not yet produced laser output in HCFGL. The associated pump wavebands and transition levels are indicated^80^

As discussed in the Introduction section, HCFGLs relying on population inversion primarily operate in the MIR region. MIR fiber lasers are significant for their extensive uses across multiple fields, including medicine, industrial processing, communication, and military defense^1^. In recent decades, the utilization of rare-earth-doped (RE-doped) fiber lasers have emerged as an effective means to generate MIR output. Fluoride glass fibers with low phonon energy (~510 cm^−1^) have attained an exceptionally high slope efficiency (approaching 60%)^73^, achieving records of 41.6 W at 2.8 μm^74^, 15 W at 3.55 μm^75^, and 2 W at 3.8 μm^76^. Utilizing chalcogenide glass fibers with reduced phonon energy (~200 cm^−1^), RE-doped fiber lasers have achieved a laser output of 0.15 W in the 5 μm waveband^77^. Nonetheless, the robustness of these soft glass fibers is significantly lower than that of silica fibers, therefore limiting the ability of soft glass fiber lasers to achieve power scaling and consistent output over prolonged durations. The problem with silica fiber is its high phonon energy (~1100 cm^−1^), resulting in significant transmission losses in the MIR band and rapid multi-phonon relaxation of excited states^78,79^. Figure 2a shows the current research progress of RE-doped fiber lasers and HCFGLs in the MIR band and the attenuation of bulk silica glass.

In contrast to RE ions, HCFGLs have the advantage of employing a diverse of gases, including a wide range of wavelength bands, hence facilitating the generation of wavelength outputs that are challenging to attain with RE-doped fiber lasers, such as the 4 μm waveband. Moreover, silica-based HCF preserves the robustness of solid-core silica fiber to some degree and demonstrates enhanced thermo-mechanical capabilities compared to soft glass fiber. This enhances the power scaling and ensures long-term consistent output of fiber lasers. These benefits enable HCFGLs to potentially exceed RE-doped fiber lasers in the MIR band. Figure 2b shows the candidate gas molecules that can be used for HCFGL in the MIR band and the related parameters.

**Fig. 3:**
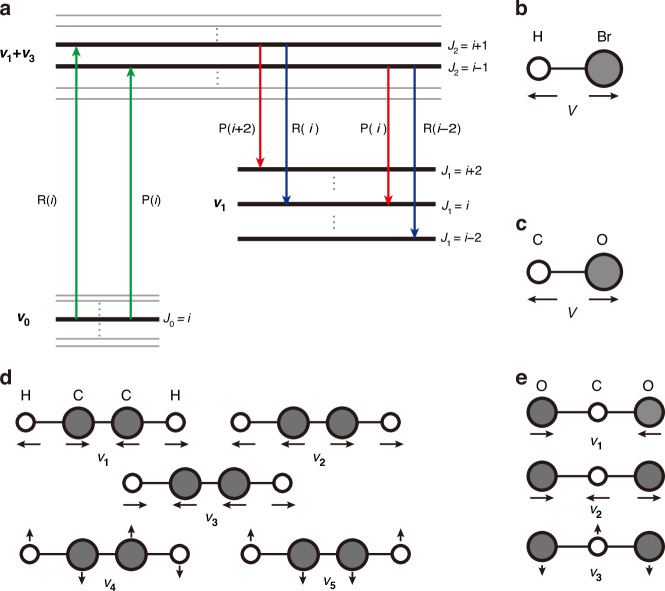
Mechanism of population inversion in HCFGL. **a** The schematic energy level diagram illustrates the molecular absorption transition and the associated laser transition. The transition mechanism of other molecules is analogous; the distinction resides in the specific upper and lower energy levels. Therefore, the C_2_H_2_ molecule is utilized as an example here. The vibrational modes of **b** HBr, **c** CO, **d** C_2_H_2_, and **e** CO_2_

Gas molecules exhibit both vibrational and rotational motions, with distinct vibrational states denoted by the vibrational quantum number *V*. A diatomic molecule, such as HBr and CO possesses a singular vibrational mode as shown in Fig. 3b, c. The vibrational quantum number *V* begins at zero and progresses through incrementally larger integers, representing a sequence of vibrational states. Each vibrational state induces a sequence of rotational states, denoted by the rotational quantum number *J*. The two collectively form the energy level structure of gas molecule. Polyatomic compounds like CO_2_ and C_2_H_2_ possess a greater number of vibrational modes. CO_2_ possesses three fundamental vibrational modes: *V*_1_, *V*_2_, and *V*_3_. C_2_H_2_ possesses five vibrational modes, designated as *V*_1_ through *V*_5_.

Take C_2_H_2_ as an example to examine the energy level transition, as shown in Fig. 3a. No appropriate pump source exists for the wavelength associated with the fundamental absorption of the C_2_H_2_ vibration mode. Therefore, the pump mechanism between the ground state (*V* = *V*_0_) and the upper level (*V* = *V*_1_ + *V*_3_) is deliberately chosen to enable absorption in the 1.5 μm waveband^80^. The population in the lower vibrational state (*V* = *V*_1_) of C_2_H_2_ is considered negligible because of the substantial energy gap between the *V*_0_ and *V*_1_ states. The population redistribution from the lower state to the ground state primarily occurs via non-radiative relaxation^81^. The pump process involves a transition from a specific rotational state (*J*_0_ = *i*) in the ground state to the rotational state (*J*_2_ = *i* ± 1) in the upper energy level. According to the quantum mechanical principle of selection, a transition to *J*_2_ = *i* + 1 constitutes an R-branch transition (Δ*J* = *J*_2_ − *J*_0_ = + 1), whereas a transition to *J*_2_ = *i* − 1 represents a P-branch transition (Δ*J* = *J*_2_ − *J*_0_ = − 1). The laser process involves a transition from the rotational state (*J*_2_ = *i* ± 1) of the upper level to the rotational state (*J*_1_ = *i*, *i* ± 2) of the lower level. Similarly, if Δ*J* = *J*_2_ − *J*_1_ = + 1, it constitutes an R-branch transition; conversely, if Δ*J* = *J*_2_ − *J*_1_ = − 1, it constitutes a P-branch transition.

### Acetylene

In comparison to other gas molecules, the absorption band of C_2_H_2_ is located in the communication band, where pump sources are readily accessible. The emission band is centered at ~3.1 μm, a spectral region characterized by relatively low attenuation, thereby enabling the realization of low-loss HCFs within this wavelength range. Consequently, the investigation of C_2_H_2_-filled HCFGL has garnered interest. The initial report of C_2_H_2_-filled HCFGL was made by Jones et al.^17,82^. Initially, the high transmission loss (20 dB/m) of the PBG-HCF utilized in the 3 μm waveband resulted in a slope efficiency of ~1% for the system. Upon substitution with the low-loss Kagome HCF, the efficiency rapidly increased to 20%^7,83^. Subsequently, the investigation of C_2_H_2_-filled HCFGL was conducted more thoroughly, resulting in continuing enhancements in output power and efficiency^17,82,84^. Furthermore, it demonstrated that HCFGL can proficiently achieve high-beam-quality output^85^.

The first research on the watt-level C_2_H_2_ laser was reported by Xu et al.^81^. The maximum continuous output power of 1.12 W was achieved with a low-loss ice-cream AR-HCF. Substantial power scaling was predominantly accomplished through advanced thermal management strategies^86–88^.

**Fig. 4:**
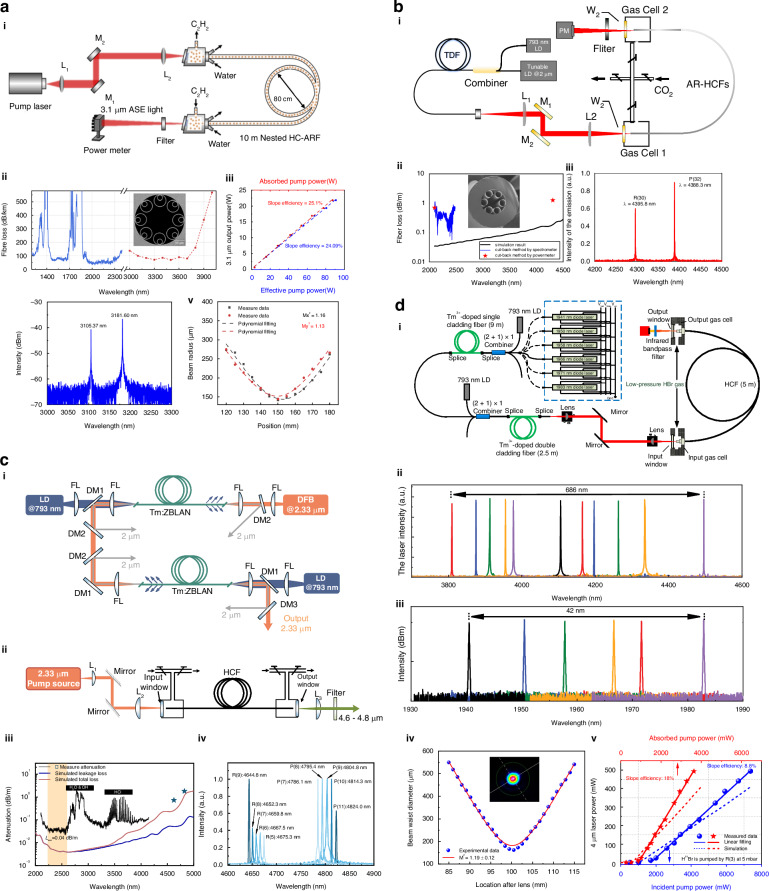
Various population inversion types of HCFGLs for different gases. **a** High-power C_2_H_2_-filled HCFGL. i The thermal effect is regulated by water cooling and gas backfilling. High power output (iii) was attained through the utilization of a NANF (ii) with low transmission loss. The output spectrum (iii) includes both P- and R-branch transitions of C_2_H_2_ and preserves high beam quality (iv). Adapted with permission from ref. ^89^ © Optical Society of America. **b** The first CO_2_-filled HCFGL. Reprinted with permission from ref. ^24^ © Optical Society of America. i The single-pass configuration. The 4.3 μm output (iii) was attained through the utilization of a 6-hole nodeless AR-HCF (ii). **c** i The first CO-filled HCFGL and the laser was pumped by a homemade 2.3 μm fiber laser. Reprinted with permission from ref. ^26^ © Springer Nature. iii The 8-hole nodeless AR-HCF exhibited a low loss in both the pumping and laser wavebands. iv The output wavelength was increased to 4.82 μm. **d** i Experimental configuration for HBr-filled HCFGL. Reprinted with permission from ref. ^25^ © Springer Nature. A 2 μm step-tuned pump source (ii) with a spread of 42 nm facilitated extensive step tuning at 686 nm inside the 4 μm band (iii) while preserving excellent beam quality (iv) and 500 mW power (v)

Recently, Song attained a continuous output of 21.8 W with an 8-hole NANF, exhibiting a beam quality of ~1.1^89^. Both the input and output terminals were cooled by water, and using back-end gas filling, as shown in Fig. 4a. This is the highest output power of C_2_H_2_-filled HCFGL gas lasers to date, as well as the highest output power of HCFGL utilizing population inversion.

Leveraging the benefits of HCF, HCFGL can attain a sufficiently high gain using a single-pass configuration; thus, the above-mentioned works were entirely predicated on this structure. Variations in cavity design will achieve different characteristics.

The ring cavity constructed using two different HCFs can significantly reduce the laser threshold to merely 16 mW under continuous pumping^90^. The amplifier structure can eliminate the threshold while simultaneously increasing efficiency. This configuration utilized a C_2_H_2_-filled HCFGL as a seed laser for the second-stage C_2_H_2_ laser^91^. The incorporation of a seed source effectively eliminated the lasing threshold and improved the efficiency, with these effects becoming particularly pronounced under high-pressure conditions. In a linear resonant cavity derived from the traditional single-pass structure, the researchers observed the self-pulsation phenomenon in HCFGL for the first time^92^.

Another direction of investigation is the all-fiber structure HCFGL. Fusion splicing is a technique employed to create an all-fiber structure in solid-core fiber lasers. Nonetheless, this poses further challenges for HCF, particularly for HCF utilized in the MIR band. This is due to the fact that achieving reduced loss in the MIR band often necessitates a larger core diameter for HCF, ranging from tens to hundreds of micrometre^11^. Selecting a solid-core fiber that matches it is challenging, and attaining low-loss fusion splicing is also difficult. To this end, Huang et al. exhibited an efficient method for attaining low-loss light coupling from solid-core fibers to AR-HCFs through fiber tapering^93^. Subsequently, Huang and Cui employed this technology alongside UV-curing adhesive to create an all-fiber structure, achieving both pulse^94^ and continuous output^95^, respectively. The average power is approximately a few hundred milliwatts. In comparison to spatial coupling, the all-fiber construction offers greater compactness and stability. The seeds utilized in the aforementioned C_2_H_2_ amplifier^91^ were likewise derived from this solution.

### Carbon dioxide

Common CO_2_ lasers mainly operate at 10.6 μm, while the absorption and emission cross-sections associated with the 4 μm (2*V*_1_ + *V*_3_ → 2*V*_1_) are significantly lower by more than an order of magnitude compared to those at 10.6 μm^7,80^, making the attainment of laser output typically challenging. The advent of low-loss AR-HCF facilitates this possibility. In 2019, Cui et al. reported CO_2_-filled HCFGL, which for the first time produced a continuous output of fiber laser above 4 μm, with a maximum output power of approximately 80 mW^24^, as shown in Fig. 4b. The pump source utilized in this system was a 2 μm single-frequency fiber amplifier, which is precisely aligned with the *V*_0_ → 2*V*_1_ absorption band of CO_2_ gas. Subsequent reports detailed the work on densely step-tunable output^96^ and pulse output^97^. Recently, a CW high-power CO_2_-filled HCFGL with an output of 6.6 W^98^ has been successfully demonstrated, utilizing a low-loss 8-tube NANF and implementing thermal management strategies comparable to those used in C_2_H_2_ laser system^89^.

Wang et al. investigated CO_2_-filled HCFGL in an amplifier configuration^99^, similar to that of C_2_H_2_ lasers^91^. In contrast to C_2_H_2_, the influence of seed injection on CO_2_ power enhancement is minimal. A slight enhancement in output power was attained by reducing the threshold. Nonetheless, due to the significantly greater emission cross-section (tenfold that of C_2_H_2_)^7^, the threshold for CO_2_ lasers is inherently low. When relaxation lines were generated, the injected seed laser actually reduced the power. Moreover, the researchers identified the self-absorption phenomena of CO_2_, whereby the injected seed laser was reabsorbed by the CO_2_ gas. The 2*V*_1_ + *V*_3_ → 2*V*_1_ radiation transition of CO_2_ is located near the *V*_0_ → *V*_3_ transition. Therefore, radiation lines that are too close to the ground state absorption line, such as R(16), will be repeatedly absorbed. The intensity of the self-absorption is also affected by pressure and temperature but ultimately depends on the wavelength separation.

### Hydrogen bromide

HBr (*V* = 2 → 1) is another gas capable of producing 4 μm radiation. Similar to CO_2_, the absorption band of HBr resides in the 2 μm waveband (*V* = 0 → 2)^80^. The energy level configuration of HBr is sparse, resulting in significant intervals between each emission line, hence including the 3.8–4.5 μm range. This facilitates the attainment of a wide range of tuned output.

In 2022, Zhou et al. first revealed HBr-filled HCFGL^25^, as shown in Fig. 4d. To illustrate the extensive wavelength tunability, the pump source was a 2 μm Tm-doped fiber amplifier utilizing a series of diode lasers, capable of encompassing the six absorption lines of the isotopes H^79^Br and H^81^Br. Eleven laser lines were identified in the investigation, comprising five R branches and six P branches. The output band was spanned from 3810 to 4496 nm, encompassing a total of 686 nm. Benefiting from molecular transition selection rules, each individual output wavelength demonstrates exceptional stability, exhibiting a linewidth under 100 MHz. Zhou et al. subsequently enhanced the pump source system, attaining a constant output of 3.1 W^100^ and a broad adjustable pulsed output^101^.

A multi-level system model developed for HBr-filled HCFGL simulates the laser performance under both continuous and pulsed pumping conditions^102,103^. Simulation results indicate that HBr pressure and fiber length are critical factors influencing laser output, threshold, and residual pumping. In the case of pulse pumping with pulse widths in the tens of nanoseconds, rotational relaxation is unobservable due to insufficient time for the higher energy level population to redistribute among other rotational levels. The gain of the gas medium is predominantly focused at the input end, leading to considerable thermal impacts and substantial population depletion.

### Carbon monoxide

Electric discharge CO lasers emerged in the 1960s, exhibiting broadband output in the 5–6 μm range, achieving efficiencies of up to 50%^104^ and continuous outputs of up to 200 kW^105^. In 1998, O. Schulz et al. first realized laser output by optically pumping CO lasers without the aid of electrical excitation^106^. Nevertheless, the CO-filled HCFGL has not been documented for an extended period, partly due to the excessively prolonged wavelength. The emission intensity of CO molecules was illustrated in the wavelength region of 4.5–5 μm (*V* = 2 → 1)^80^. Although advanced HCFs can reduce the overlap between material and mode fields to ~10^−5^, the transmission loss of HCFs remains influenced by the considerable loss of silica materials in this range^11^. The curve depicted in Fig. 2a indicates a substantial increase in the loss of bulk silica glass when the wavelength exceeds 4.6 μm. The loss attains a value of 13,000 dB/m at a wavelength of 4.8 μm^11,107^. In 2012, Jones et al. filled CO into a silver-coated capillary and utilized an OPO as a pump source to achieve the R(7) line at 4.67 μm and the P(6) lines at 4.78 μm^7,83^. However, no power measurements were reported in this work. The recent advent of low-loss AR-HCF has enabled the extraction of laser output from CO gas in HCF. Another obstacle in advancing CO laser technology lies in the development of a suitable pump system. This is primarily due to the first overtone absorption band (*V* = 0 → 2) of CO is centered at 2.3 μm, a spectral region where the availability of high-power, narrow-linewidth fiber laser sources remains extremely limited.

In 2024, Li et al. reported a 4.8 μm CO-filled HCFGL^26^, as shown in Fig. 4c. The laser was produced using low-loss AR-HCF with measured losses of 0.73 dB/km at 4.64 μm and 1.81 dB/km at 4.82 μm. A maximum MIR output power of 46 mW and a tuning range of 180 nm (from 4644 to 4824 nm) were achieved utilizing a homemade 2.33 μm narrow linewidth fiber laser^108^. This demonstration signifies the longest-wavelength silica-based fiber laser to date. Additional wavelength expansion may be attained by altering the pump absorption line and refining the laser structure.

### Other gases

Besides the above-mentioned typical gases, there have been reports of HCFGL using gases such as HCN, N_2_O, and I_2_. HCN exhibits absorption and emission bands similar to those of C_2_H_2_, achieving pulsed output with an energy of 56 nJ^7,83^. The absorption band of N_2_O for pumping is located at 1.5 μm telecom band, while its emission band is centered at 4.6 μm. By employing an OPO as the pump source, researchers have successfully demonstrated 4.6 μm pulsed laser emission with an output energy of 75 nJ^23^. Through optical pumping with a 532 nm Nd laser, I_2_ has demonstrated laser emission in both visible^109^ and NIR^110^ regions within a Kagome HCF, achieving output power levels approaching 9 mW. This achievement establishes I_2_ as the sole gas in population-inversion-based HCFGL that operates outside the MIR region.

### Brief summary

The C_2_H_2_-filled HCFGL is the most extensively researched type of population inversion HCFGL. This is due to the readily accessible 1.5 μm pump sources and the advancement of low-loss HCF. Since its inception in 2010, the output power and efficiency have been consistently enhanced, achieving 21.8 W. Given the advent of more advanced HCFs and enhancements in laser structure, this record is unlikely to endure for an extended period, and there is optimism that it will exceed the 41.6 W landmark established by the Er^3+^ fiber laser^74^ in the comparable spectrum. Despite HCN gas exhibiting a comparable spectrum and cross-section to C_2_H_2_, its extreme toxicity renders it a challenging substitute for C_2_H_2_. The primary emphasis in the 4 μm band should be directed towards CO_2_ and HBr for an extended duration. Despite being more expensive than 1.5 μm lasers, high-power 2 μm lasers are more readily accessible. CO gas can provide longer wavelength output; nevertheless, this imposes greater demands on low-loss HCF. Furthermore, the requisite 2.3 μm pump source is exceedingly rare and lacks a reliable solution. This can potentially be achieved through high-order overtone absorption of CO gas for NIR band pumping^26^.

## HCFGLs based on simulated Raman scattering

HCFGLs based on SRS have emerged as a novel light source in tandem with the advancement of HCFs, utilizing the SRS of gas molecules within the HCFs to facilitate laser wavelength conversion. Unlike HCFGLs based on population inversion, the SRS-based HCFGLs are unlimited by the specific wavelength of the pump laser. Theoretically, the generation of Raman lasers at arbitrary wavelengths can be achieved through the strategic selection of pump sources with appropriate wavelengths and HCFs featuring suitable guidance bands.

**Fig. 5:**
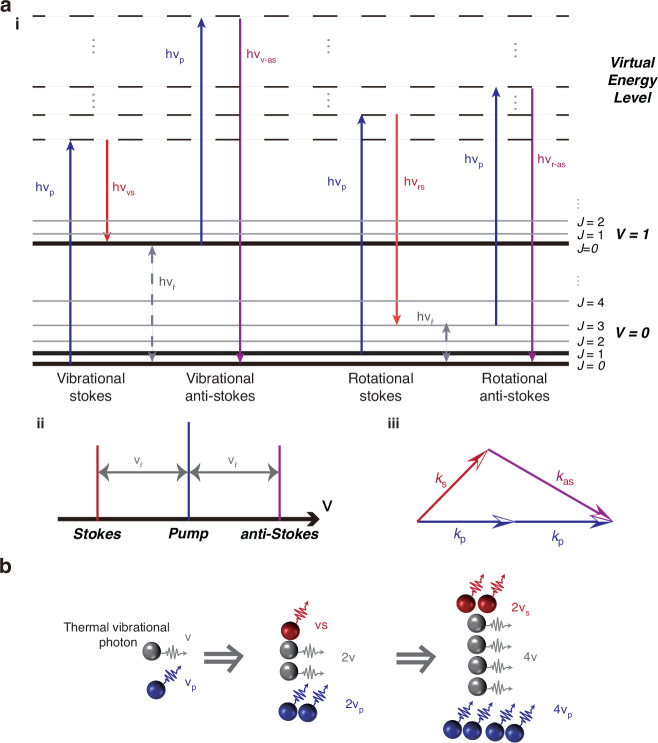
The schematic diagram of the principle of HCFGLs based on SRS. **a** i Schematic energy level diagram describing the molecular transition for rotational SRS, vibrational SRS and the corresponding anti-Stokes scattering. Here, *v* is frequency, *h* is Planck constant, and *v*_R_ represents the phonon frequency that corresponds to the transition between different vibrational or rotational energy levels. ii Frequency conversion relationship between Stokes, pump and anti-Stokes. iii The schematic diagram illustrates phase matching during the anti-Stokes process, and *k* represents the wave vector. **b** A schematic illustration depicting the Stokes laser (in Red) amplification with the exponential-growth trend when the pump light (in Blue) is sufficiently intense

Figure 5a-i illustrates the energy-level transitions of gas molecules during the SRS process. Here, *V* denotes the vibrational energy level, and *J* represents the rotational energy level. When interacting with the pump laser, gas molecules transition from the ground state to a virtual energy level and subsequently relax to higher vibrational or rotational states, emitting Stokes photons in the process. Similarly, when the pump laser interacts with molecules in an excited state, anti-Stokes scattering occurs, following analogous energy-level transitions. The anti-Stokes scattering exhibits the same magnitude of frequency shift as Stokes scattering but in the opposite direction relative to the pump laser (Fig. 5a-ii). However, this process is more challenging to achieve due to the stringent phase-matching requirements, as illustrated in Fig. 5a-iii. When the pump photon density is sufficiently high, the Stokes photons exhibit an exponential growth trend, as depicted in Fig. 5b. The threshold power for gas Raman lasers in HCFs can typically be estimated by the following equation^111^:

1 $${P}_{{th}}=\frac{{A}_{{eff}}}{g}\frac{{{\rm{\alpha }}}_{p}\left({G}_{{th}}+{{\rm{\alpha }}}_{s}L\right)}{1-\exp \left(-{{\rm{\alpha }}}_{p}L\right)}$$

where *A*_*eff*_ represents the effective mode area of the optical field, *g* denotes the Raman gain coefficient, *α*_*p*_ and *α*_s_ are the transmission losses of the pump and Stokes light in the fiber, respectively, *L* is the fiber length, and *G*_*th*_ represents the net gain factor at threshold. By optimizing the fiber geometry and attenuation as well as enhancing the gas Raman gain, the required threshold power can be effectively reduced.

Currently, SRS-based HCFGLs have employed various gases, including hydrogen (H_2_), methane (CH_4_), deuterium (D_2_), ethane (C_2_H_6_), nitrogen (N_2_), oxygen (O_2_), carbon dioxide (CO_2_), carbon tetrafluoride (CF_4_), and sulfur hexafluoride (SF_6_), etc., as Raman media. These gases have enabled laser emissions across the ultraviolet (UV) to MIR spectral regions. Among these gain media, H_2_, CH_4_, and D_2_ have garnered more extensive research attention due to their high Raman gain and large Raman frequency shifts. A high gain coefficient implies that the generation of Stokes lasers is more straightforward, while a significant Raman frequency shift aids in the effortless generation of mid-to-far infrared laser emissions. Table 2 depicts the frequency shift coefficients and relative scattering cross-section for the aforementioned gas Raman gain media^112,113^, along with the first-order Stokes and anti-Stokes laser wavelengths that can be produced by these gases under pump radiation at 1064 nm, 1550 nm, and 1950 nm.

**Table 2:** Spectroscopic information on the main gas molecules

Gas molecules	Coefficient of Raman frequency shift /cm^−1^	Relative scattering cross section (related to nitrogen)	*λ*_p_^a^ = 1064 nm				*λ*_p_ = 1550 nm				λ_p_ = 1950 nm			
			*λ*_s1_^b^	*λ*_as1_^c^	*λ*_s2_	*λ*_as2_	*λ*_s1_	*λ*_as1_	*λ*_s2_	*λ*_as2_	*λ*_s1_	λ_as1_	λ_s2_	λ_as2_
H_2_	4155	3.9	1907	738	9187	565	4354	943	/^d^	677	10,275	1077	/	744
	587	1.6	1135	1001	1216	946	1705	1421	1895	1311	2202	1750	2529	1587
D_2_	2987	3.9	1560	807	2920	651	2886	1059	20,937	805	4670	1232	/	901
	415	1.6	1113	1019	1167	978	1657	1456	1779	1373	2122	1804	2327	1678
CH_4_	2917	8.6	1543	812	2805	656	2829	1067	16,191	814	4522	1243	/	912
C_2_H_6_	2954	15	1552	810	2865	653	2859	1063	18,395	809	4599	1237	/	906
CO_2_	1389	1.1	1249	927	1510	821	1975	1275	2722	1083	2674	1534	4255	1265
SF_6_	775	3.9	1178	970	1319	892	1804	1359	2157	1210	2370	1657	3019	1440
CF_4_	908	1.2	1160	983	1274	913	1762	1384	2040	1250	2297	1694	2795	1497
N_2_	2331	1	1415	853	2111	711	2427	1139	5588	900	3575	1341	21450	1021
O_2_	1550	1	1274	913	1588	800	2040	1250	2984	1047	2795	1497	4930	1215

^a,b,c^The wavelength of the pump, Stokes, and anti-Stokes

^d^The SRS process cannot be realized due to the pump energy level limitation

### Hydrogen

In gas-based SRS, H_2_ is recognized as one of the most commonly used gaseous media. As the lightest diatomic molecule, H_2_ has a relatively simple vibrational-rotational energy level structure and exhibits significant energy level spacing. Besides, H_2_ exhibits the highest Raman gain and the largest Raman frequency shift. This characteristic facilitates the achievement of wide-range. Studies on H_2_-filled HCFGL have been extensively reported across the UV, visible, and NIR to MIR spectral regions.

**Fig. 6:**
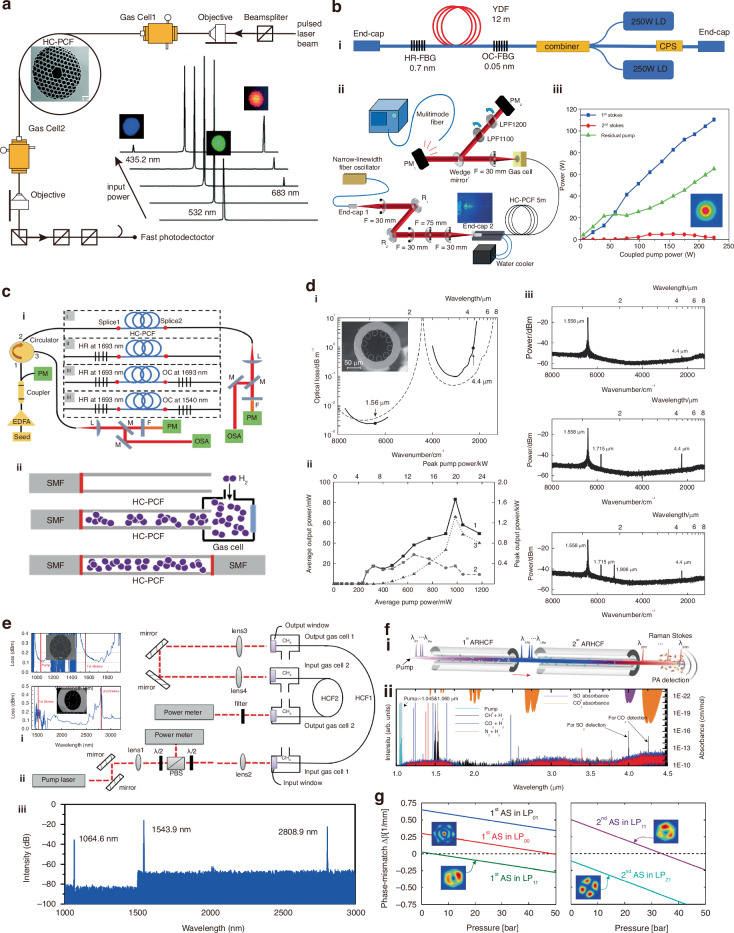
Schematics and results of the representative HCFGL in SRS. **a** The first HCFGL based on SRS in 2002. Modified from F. Benabid et al., Science, 10.1126/science.1076408 [2002], AAAS^4^. **b** The 110 W HCFGL via SRS. i Self-developed MOPA structure 380 W high-power pump source. ii The details of the experimental configuration. iii The power characteristics of the 110 W Stokes laser. Reprinted with permission from ref. ^119^ © Chinese Laser Press. **c** i Typical HCFGL based on SRS with all-fiber structure. Reprinted with permission from ref. ^128^ © Optical Society of America. ii The fabrication process of all-fiber gas cavity. **d** The first 4.4 μm HCFGL based on SRS. i The transmission loss spectrum of the employed HCF and a scanning electron micrograph (SEM) of the cross-section. ii The power characteristics of the 4.4 μm Stokes laser. iii The output spectrum at different peak pump power. Reprinted with permission from ref. ^18^ © Turpion Ltd. **e** The first HCFGL with cascaded structure. i The transmission spectrum of the HCFs and their SEMs of the cross-section. ii The experimental configuration. iii Output spectrum. Adapted with permission from ref. ^139^ © Optical Society of America. **f** i Concept of synthesizing multiple spectral lines. ii The output spectrum under different gas combinations as well as the absorption spectrum of the CO_2_ and SO_2_. Reprinted with permission from ref. ^140^ © Springer Nature. **g** The phase mismatch for the first and second anti-Stokes laser at different gas pressures and various pump modes. Reprinted with permission from ref. ^144^ © Optical Society of America

In 2002, Benabid et al. reported a H_2_-filled HCFGL, marking a historical milestone as the first HCFGL based on SRS^4^. They achieved a Raman laser output at 683 nm based on a single-pass structure, as shown in Fig. 6a. This study highlighted the pivotal role of HCFs in reducing the Raman threshold and enhancing the Raman conversion efficiency, thereby ushering in a new era for HCFGLs.

In comparison to the light source operating in the visible spectrum region, high-power pump sources at 1 μm are more readily available. Therefore, HCFGLs based on SRS that are pumped by 1 μm lasers have been extensively studied. The output wavelengths predominantly fall within the 1.1 μm and 1.9 μm bands, which align with the rotational and vibrational Stokes lines of H_2_, respectively.

In 2004, Benabid and colleagues reported the first study of pure rotational SRS of H_2_ within a HCF^111^. Their research achieved a quantum efficiency of 92% for the laser output at 1135 nm, reducing the required pump pulse threshold energy by a factor of 10^6^. By increasing the energy of the injected pump pulses, more cascaded rotational Stokes lines can be obtained within a PBG-HCF^114^. These works confirmed the unique advantages of HCFs in gas SRS, making flexible wavelength conversion within HCFs a reality. To further enhance the stability of gas cavities, Benabid et al. constructed an all-fiber gas cavity by splicing PBG-HCFs with single-mode solid-core fibers^115^. By constructing a resonant cavity with fiber Bragg gratings (FBGs), the conversion can be further enhanced by more than 30 dB^116^.

Benefiting from the high gain coefficient, rotational Stokes laser in H_2_-filled HCFs can also be achieved with CW pumping. In 2007, Couny et al. realized the first CW-pumped fiber gas Raman laser source, achieving a power conversion efficiency of 45%^117^. Subsequently, by constructing resonant cavitie^117^, using high-power pump sources^118,119^, and employing longer fibers^120^, low-threshold high-power 1.1 μm Stokes lasers have been obtained. Notably, based on a high-power pump source with a master oscillator power amplifier (MOPA), as shown in Fig. 6b, Cui et al. achieved 110 W Raman laser at 1153 nm. This represents the highest output power of SRS-based HCFGL.

Wang er al. reported the first 1.9 μm HCFGL in 2014^121^. A linearly polarized 1064 nm pulsed laser was used to pump a 6.5 m H_2_-filled AR-HCF, obtaining a vibrational Stokes laser at 1908 nm. The system exhibited a quantum efficiency of 48% with a peak power exceeding 2 kW. By increasing pump power and optimizing HCF parameters, researchers managed to achieve 1.9 μm Stokes laser with peak powers in the hundreds of kilowatts^122,123^. In 2018, Li et al. realized the first H_2_-filled Raman amplifier by injecting a 1.9 μm seed light into the HCF^124,125^. The results indicated that the injection of seed light played a crucial role in reducing the threshold power.

Additionally, by utilizing rotational SRS of H_2_ with a 1.5 μm pump source, Raman laser emissions at 1.7 μm can be achieved. In 2020, Huang et al. reported the first fiber H_2_ Raman laser source in this band^126^. They employed a tunable 1.5 μm pulsed amplifier as the pump source to generate tunable pure rotational Stokes laser emissions in the range of 1687–1723 nm. Subsequently, researchers investigated the output characteristics of all-fiber gas Raman oscillators^127–129^. Through discharge splicing and incorporating FBGs, low-threshold watt-level laser with both CW and pulsed pumping have been achieved. The typical structure and the fabrication process are shown in Fig. 6c. Notably, for all-fiber HCFGLs with pulsed pumping, the Raman threshold can be significantly reduced when the repetition frequency of the pump pulses matches the resonant frequency of the cavity^129^.

Pump sources at the 1.5 μm band can also be utilized for the vibrational SRS of H_2_, enabling the generation of MIR laser emissions beyond 4 μm. Based on a 15-m-long AR-HCF, Gladyshev et al. achieved the first pulsed fiber Raman laser output at 4.42 μm pumping with a pulsed Er-doped fiber amplifier at 1558 nm^18^. Its output characteristics are shown in Fig. 6d, exhibiting an average power of 30 mW and a quantum efficiency of about 15%. By optimizing the performance of the pump source and ensuring linearly polarized output, the average power^19^ and pulse energy^130^ of the 4 μm Stokes laser have been substantially improved. For the UV spectrum region, H_2_-filled HCFGLs are predominantly generated as optical frequency combs^45,131,132^. Among the research efforts, Couny and colleagues have achieved 45 Stokes lines^45^. Multiple vibrational and rotational Stokes lines were obtained spanning a range from 325 to 2300 nm.

### Alkanes

Alkanes rank as the most commonly used Raman medium gases following H_2_. Unlike other gas molecules, alkane molecules are less susceptible to rotational SRS, meaning that the resulting spectral output usually comprises only pure and singular vibrational Stokes lines. As such, for prevalent alkanes like CH_4_ and C_2_H_6_, the conversion efficiency of their first-order vibrational Stokes lasers is expected to surpass that of H_2_.

In 2016, Chen et al. reported the first HCFGLs based on SRS of alkane^133^. Employing a 1064 nm microchip laser as the pump source, they achieved a vibrational Stokes laser output at 1553 nm within a C_2_H_6_-filled AR-HCF. The peak power of the output exceeded 400 kW.

The study of HCFGLs based on CH_4_ came in 2017. Chen et al. achieved a vibrational Raman laser output at 1544 nm within a AR-HCF filled with CH_4_^134^. The power conversion efficiency was 66.4% with a remarkable quantum efficiency of 96.3%. By increasing pump power, Li et al. achieved a Stokes laser output at 1544 nm with an average power of 0.83 W^135^. Recently, the output power of CH_4_-filled HCFGLs in the 1.5 μm band has been elevated to the watt level^136,137^. When an appropriate HCF is utilized in conjunction with a tunable pump source operating at 1.5 μm, a tunable Stokes laser output at 2.8 μm can be also realized^138^.

Stokes pulses generated by CH_4_-based HCFGLs can also be utilized for the generation of MIR pulses. Li et al. first adopted a cascade approach to achieve the laser conversion from 1 to 2.8 μm^139^, as shown in Fig. 6e. The first stage of the system utilized a 1064 nm pulsed laser to pump a CH_4_-filled AR-HCF, generating a 1544 nm Stokes laser output. Taking the first stage’s Stokes laser as the pump source, the second stage achieved the laser at 2809 nm based on a another CH_4_-filled AR-HCF. The power of the 2.8 μm laser is 13.8 mW with an overall system quantum efficiency of 65%. This study pioneers a robust demonstration of the viability of a multi-stage cascaded structure for HCFGLs. Recently, Wang et al. employed Yb-doped fiber lasers at 1044 nm and 1060 nm, leveraging the cascaded SRS effect of CH_4_ and H_2_ as illustrated in Fig. 6f, to obtain multi-wavelength MIR laser outputs at 3.99 μm and 4.25 μm^140^. By adjusting the wavelength of the 1 μm pump source, the MIR laser wavelength can be effectively tuned to match the absorption lines of gases such as SO_2_ and CO_2_. This capability is important for applications such as gas sensing.

Solid-state lasers at 1 μm with peak power exceeding megawatt level have also been extensively applied in CH_4_-filled HCFGLs as the pump source. MIR Stokes lasers can be directly generated within a single HCF in this way. In 2018, Cao et al. employed a self-developed picosecond-level Nd: YAG laser at 1064 nm with a peak power of 91 MW as the pump source^141^. They obtained the Stokes laser at 2812 nm based on a 3 m HCF filled with 1.8 MPa CH_4_, achieving an output average power of 113 mW and a quantum efficiency of approximately 40%. This marked the first fiber-based gas Raman laser with high peak power in the MIR region. Subsequently, by optimizing the pump source, the output power has been enhanced to the watt level^142,143^. Additionally, some anti-Stokes lines can also be observed when employing these high-peak-power pump sources, albeit with relatively low intensities. Trabold et al. have noted that integration of fundamental and higher-order mode pumping, in conjunction with the meticulous control of gas pressure (Fig. 6g), is instrumental in achieving the phase matching required for the production of anti-Stokes lasers^144^.

Currently, HCFGLs based on alkane SRS have not yet achieved CW Stokes laser emission. This is attributed to the high thresholds for vibrational SRS. The Raman frequency shifts corresponding to vibrational SRS in alkanes are significant, necessitating a low-loss HCF with wide transmission windows. Previously, HCFs meeting these criteria have typically featured core diameters exceeding 30 μm, which has conventionally led to vibrational SRS threshold powers in the several-kilowatt range or higher. The realization of CW output is expected by constructing a high-power CW pump source, reducing the core diameter of HCF, and optimizing the fiber length.

### Deuterium

As an isotope of H_2_, D_2_ is also a familiar Raman medium. The molecular mass of D_2_ is approximately twice that of H_2_, resulting in a smaller Raman frequency shift. Additionally, compared to H_2_, the threshold power for SRS in D_2_ is higher, and it is more likely to generate multiple rotational Raman spectral lines.

Similar to the characteristics of CH_4_, the generation of 1.5 μm vibrational Stokes laser inside a D_2_-filled HCF can be achieved when employing pump light at 1 μm. Pei et al. demonstrated near-single-mode, narrow-linewidth laser emission at 1560 nm based on a self-developed 1064 nm pulsed fiber laser^145^. By optimizing the incident angle of the pump light, the maximum Stokes power exceeded 7 W, accompanied by excellent beam quality.

The generation of 2.9 μm D_2_ vibrational Stokes laser has also attracted significant attention when pumped by 1.5 μm laser sources. Gladyshev et al. documented an exploration involving a mixture of H_2_ and D_2_ within a HCF^146^. They employed a 1558 nm Er-doped fiber amplifier to pump an 11 m HCF charged with a gas mixture, resulting in Stokes laser emissions at 2.9 μm, 3.3 μm, and 3.5 μm. By refining the characteristics of the pump source and the coupling structure, Huang et al. enhanced the tunable Stokes laser output to a power of 6 W across the range of 2851–2921 nm^147^.

By employing a cascaded pumping technique, MIR laser emission via D_2_ SRS can be effectively produced. Huang et al. initiated the process by employing a 1 μm pump light to generate a 1.5 μm vibrational Stokes laser in D_2_^148^. The output was then employed to drive a second-stage HCF filled with CH_4_, thereby yielding cascaded Stokes laser emissions at 2.8 μm.

Harnessing a 1.5 μm laser as the pump and exploiting the rotational SRS in D_2_, the generation of Stokes laser radiation at 1.6 μm becomes feasible. In 2019, Cui et al. utilized a custom-developed tunable 1.5 μm pulsed pump source^149^. Within a commercial PBG-HCF, they succeeded in producing rotational Raman laser emissions spanning 1640 to 1674 nm, reaching an average power of about 0.8 W and a maximum power conversion efficiency of 60%. Following this, through optimization of the pump source and the fiber length, they managed to elevate the output power to a remarkable 2.91 W^150^.

### Other gases

Beyond the gases previously discussed, a sparse array of research has also emerged detailing the construction of HCFGRL based on SRS with alternative gases.

Edelstein et al. conducted a groundbreaking investigation into the SRS involving SF_6_ and CF_4_ gases within the HCFs^151^. Employing a tunable ytterbium-doped fiber laser operating at 1030 nm as the pump, they successfully generated a vibrational Stokes laser at 1119 nm within a 15 m HCF pressurized with 1.2 MPa SF_6_, resulting in an impressive power conversion efficiency of 55.7%. When the gas was switched to CF_4_ at the same pressure, a 1136 nm Stokes laser with a power conversion efficiency of 45.4% can be obtained. First-order Stokes laser at 1248 and 1276 nm can be also achieved in HCFs filled with CO_2_^152^ and O_2_^153^, respectively, when pumped with a 1064 nm laser. Recently, an N_2_-filled HCFGL has also been achieved with high energy^154^. By using a custom 1060 nm fiber laser, Hong achieved a Stokes laser at 1407 nm within a 16-m HCF filled with 15 bar N_2_. The Stokes pulse obtains a width of 3.3 ns with 26.5 μJ pulse energy. This study reaffirms the potential of HCFGL as a viable option for producing high-energy narrow-linewidth lasers that beyond the gain spectrum of RE-doped fiber lasers.

### Brief summary

**Fig. 7:**
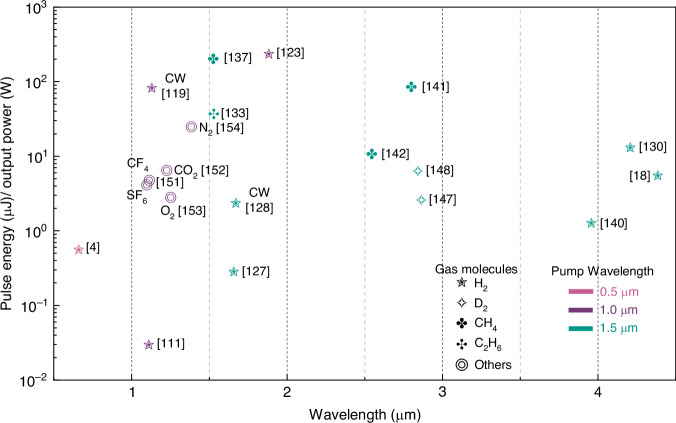
Summary of the representative HCFGLs based on SRS in terms of pulse energy or output power. Pulsed and CW outputs are both involved, with each corresponding to the pulse energy and output power on the left *y* axis, respectively^4,18,111,119,123,127,128,130,133,137,140–142,147,148,151–154^. HCFGLs in CW are labeled with “CW”. The different shapes and colors of the dots represent the gas molecules employed and the pump wavelengths, respectively, as shown in the figure. HCFGLs filled with other gases have also been labelled with their gas species

Currently, HCFGLs based on SRS have achieved Stokes laser output covering the visible to MIR spectral regions. Figure 7 summarizes the primary research accomplishments thus far. It is evident that H_2_ remains the most widely used Raman gain medium, with output wavelengths primarily located at 1.1 μm, 1.7 μm, 1.9 μm, and 4.4 μm. Owing to their robust Raman gain properties, H_2_-filled HCFGLs have successfully realized Stokes lasers under both pulsed and CW pumping. Notably, a CW Raman laser output of 110 W has been achieved, marking the highest power level yet achieved by HCFGLs. Alkanes are the next most frequently used medium, with their output wavelengths mainly concentrated at 1.5 μm and 2.8 μm. Polyatomic alkane molecules possess a complex energy level structure, which complicates the process of initiating rotational SRS, thereby presenting a greater challenge. Therefore, HCFGLs filled with alkanes typically produce only pure vibrational Raman lasers, which hold the promise of achieving conversions with higher quantum efficiency. Utilizing the injection of seed light, a peak quantum efficiency of 96% has been demonstrated, marking a remarkable milestone in the field. D_2_ shares similar characteristics to hydrogen but with lower Raman gain and nearer frequency shifts. Its output wavelengths are mainly concentrated at 1.5 μm, 1.65 μm, and 2.9 μm. Sulfur hexafluoride, carbon tetrafluoride, oxygen, and nitrogen have also been used in HCFGLs based on SRS, with output wavelengths at 1.1 μm, 1.2 μm, and 1.4 μm. Their specific characteristics still call for further investigation.

## Challenges and future prospects

With the continuous advancement of HCF, HCFGLs have garnered increasing research interest. Currently, HCFGLs have achieved laser output across multiple wavebands, including UV, NIR, short-wave infrared, and MIR regions, making them an effective means for generating lasers with unattainable wavelengths, especially in the MIR range. Furthermore, these lasers exhibit exceptional beam quality, narrow spectral linewidth, and significant potential for power scaling, making them highly promising for advanced applications in optical communications, remote sensing, and precision detection systems. Here are some prospects for the future development of HCFGLs.

### Power enhancement

Over the past two decades, the output power of HCFGLs has continuously increased, achieving outputs exceeding hundreds of watts in the NIR region^119^ and tens of watts in the MIR region^89^. This achievement primarily stems from advances in HCF fabrication techniques. The low-loss, broadband guidance of HCF provides an ideal platform for efficient light-gas interaction, while the high attenuation of higher-order modes ensures excellent beam quality in the transmitted laser. Meanwhile, high-power pump sources with superior beam quality deliver sufficient pump power while enabling highly efficient coupling. Therefore, HCFGLs hold great potential for scaling to even higher power levels.

**Fig. 8:**
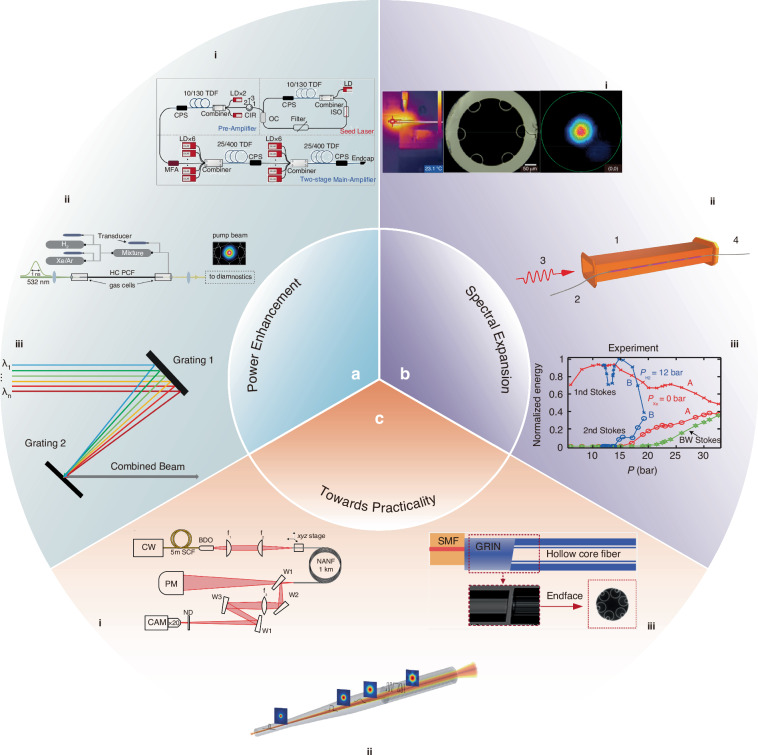
Prospect of HCFGLs. **a** i The high-power narrow-linewidth pump source with classical MOPA architecture. Reprinted with permission from ref. ^155^ © Optical Society of America. ii A representative experimental setup of a HCFGL with the buffer gases filling. Reproduced with permission from ref. ^176^ © American Physical Society. iii The schematic diagram of the spectral beam combining for multiwavelength high-power HCFGLs. **b** Spectral expansion of diverse HCFGLs. i The advanced chalcogenide AR-HCF can attain 10 W single-mode laser transmission in the MIR range. Adapted with permission from ref. ^168^ © Optical Society of America. ii Microwave discharge HCFGL that directly employs gas-filled HCFs as a laser generator. Reproduced with permission from ref. ^173^ © Institute of Electrical and Electronics Engineers. iii Buffer gases have been shown to enhance the efficiency of visible light anti-Stokes processes. Reproduced with permission from ref. ^176^ © American Physical Society. **c** i A typical high-power laser transmission configuration based on HCF. Reproduced with permission from ref. ^177^ © Springer Nature. ii Schematic diagram of reverse tapered solid-core fiber. Reproduced with permission from ref. ^180^ © American Chemical Society. iii A novel approach for reducing back reflection by combining an angle-cleaved HCF with offset-splicing the mode-field adapter to the SMF. Reproduced with permission from ref. ^182^ © American Chemical Society

The increase of the output power of pump sources characterized by a narrow linewidth, stable wavelength, and superior beam quality is of paramount importance in power enhancement of the HCFGLs, ensuring an adequate supply of pump photons during the conversion. Utilizing the classical MOPA architecture (as depicted in Fig. 8a-i^155^), the generation of high-power, narrow-linewidth CW or pulsed pump radiation is feasible through multiple filtering techniques and multi-stage amplification. This constitutes the fundamental prerequisite and a robust guarantee for power enhancement in HCFGLs.

Furthermore, the introduction of buffer gases within the gas cavity presents an intriguing avenue for exploration (Fig.8a-ii). Buffer gases hold the potential to facilitate collisional relaxation, enhance molecular mobility, improve the cavity’s heat dissipation capabilities as well as optimize its high-power performance.

In addition to the strategies above, it is feasible to contemplate the implementation of spectral beam combining techniques, as illustrated in Fig. 8a-iii. This advanced optical approach involves the use of custom-designed diffraction gratings to integrate multiple laser beams emitting at different wavelengths, thereby achieving a high-power, multi-wavelength output from HCFGLs.

### Spectral expansion

The advancement of HCFGL is intrinsically linked to high-performance HCF. The minimal overlap between material and mode domains enables silica-based HCF to exhibit low loss equivalent to that of commercial soft glass fibers in the MIR band. Nonetheless, due to the significant loss of silica glass at extended wavelengths, it remains challenging for silica-based HCF to achieve a loss below 1 dB/m^11,156–158^. For HCFGL, it is essential to consider the dual low loss characteristics of the pump and laser bands. This necessity has led to the exploration of soft glass materials with low loss in the MIR range. MIR transmitting glasses usually have inferior mechanical strength compared to silica, considerably steeper viscosity versus temperature curves, and are not available in pre-fabricated tubes. Consequently, the fabrication of preforms and mechanically robust soft-glass fibers with uniform structures (transversally and longitudinally) is noticeably challenging^159–167^. Nonetheless, chalcogenide glass HCF capable of stably transmitting 10 W in single mode has emerged^168^, as shown in Fig. 8b-i. Despite the transmission efficiency being below 20%, this nevertheless demonstrates the potential of soft glass HCFs.

Soft glass HCF may effectively broaden the low loss bandwidth of HCF to 10 μm^160^. HCFGL can attain a broader spectrum of laser output using various gasses. For instance, a laser output of 2.5–8 μm can be attained only using CO gas^169^. Nonetheless, low-loss HCF alone remains insufficient. Both population inversion and SRS are based on optical pumping, which places stringent demands on the pump light source.

It is difficult to find a suitable light source for the wavelengths corresponding to many gas absorption lines. This means that large amounts of gas or multiple transitions of gas are not feasible with current optically pumped HCFGL. This explains why only a restricted number of gases are depicted in Fig. 2b. The idea of discharged HCFGL can solve this problem by directly using HCFGL as a generator rather than a frequency converter for pump lasers, as shown in Fig. 8b-ii. Both direct current excited^170,171^ and radio-frequency excited^172^ HCFGLs have been reported, but microwave excitation was ultimately used to achieve laser output, although the power was only a few mW^173–175^. Currently, a major challenge with discharged HCFGL is how to maintain a stable gas discharge plasma in longer HCFs (multimeters). In addition, discharged HCFGL exhibits characteristics different from those of conventional gas lasers^173^. This reflects the need for further research on discharged HCFGL.

Another promising direction for wavelength expansion is towards shorter wavelengths, particularly in the UV and visible bands. Compared to the MIR region, HCF exhibits lower losses in these ranges, with the added advantage of mitigating the photon darkening effect. Achieving this through population inversion requires addressing two aspects: first, selecting a gas medium with appropriate pumping and absorption bands, and second, developing a suitable pump source. Discharged HCFGL also appears to be a promising solution in this context. HCFGL based on anti-Stokes offers the advantage of reduced requirements for pump sources. However, its efficiency remains limited due to complex nonlinear processes. Although existing studies have demonstrated that buffer gases can enhance the efficiency of anti-Stokes process (Fig. 8b-iii)^176^, further research is still necessary to fully optimize its performance.

### Towards practicality

Compared to traditional solid-core fibers, the laser energy transmitted inside the HCFs is primarily confined within the hollow core, with minimal overlap between the mode field and the capillary wall. This positions HCFGLs as promising candidates for attaining elevated levels of laser power output. In 2022, Mulvad et al. successfully transmitted a kilowatt-average-power single-mode laser over kilometer-scale HCFs^177^, and the corresponding experimental setup is shown in Fig. 8c-i. This work paves the way for high-power long-distance laser transmission in HCFs. Currently, CW power transmission up to 3 kW has been realized in HCFs^178^, further confirming the high-power output potential of HCFGLs. Additionally, in traditional lasers based on solid-core fibers, the presence of Fresnel reflections at the fusion point or output end is inevitable, which significantly impacts laser performance at high power levels. This issue does not exist for HCFGLs, as the hollow core of the HCF can effectively avoid it.

Until now, the prevailing HCFGLs predominantly feature a spatial configuration, wherein the pump light is coupled into the gas cell through free-space techniques. Streamlining the structure of HCFGLs and achieving low-loss connections between the pump pigtail and the gas cell are crucial steps toward enhancing the compactness and viability of the HCFGLs for practical applications. By selecting appropriate pigtails of pump source and optimized HCFs, and employing techniques such as tapering^93,179^ and reverse tapering approach^180^ (Fig. 8c-ii), the modal-field mismatch between solid-core fibers and HCFs can be effectively reduced, enabling low-loss fusion splicing. Currently, this method has reduced the fusion splicing loss by nearly eightfold^181^. Introducing a graded-index fiber, serving as a mode-field adapter to bridge between the SMF and the HCF, is also an effective approach which has achieved a record-low coupling loss of 0.079 dB. Due to the core refractive index mismatch, Fresnel reflection occurs at the glass-air interfaces, introducing additional insertion loss that degrades the performance of all-fiber HCFGLs. Recently, Shi et al. reported a notable fusion technology with ultralow back reflection of less than −60 dB^182^. By introducing an offset-spliced mode-field adapter, as shown in Fig. 8c-iii, the Fresnel reflection at the splice point can be effectively eliminated^182^. Furthermore, by optimizing splice parameters^183,184^ combined with thermally conductive adhesives^185,186^ or customized mechanical fixtures^183,187^, long-term thermo-mechanical reliability can be significantly improved, enhancing the high-power output performance of HCFGLs.

## Conclusion

Conventional solid-core fiber lasers, including double-clad and multicore fiber lasers, etc, have achieved significant progress^2,188–192^. While their output remains predominantly confined to the 1–2 μm band, fundamentally constrained by rare-earth ion transitions. HCFGLs offer unprecedented spectral flexibility where conventional solid-core fiber lasers face fundamental limitations. The synergistic advancement of HCF fabrication technology and pump source development enables HCFGLs to break new ground in high-power laser generation at challenging wavelengths, exhibiting superior high-power potential in UV and MIR spectral regimes.

We have reviewed the development process and major achievements of HCFGLs. Initially, we introduced an overview of the main types of HCFs and their key developmental milestones. Subsequently, we discussed the primary achievements of HCFGLs based on population inversion and those based on SRS, in accordance with their respective principles. Finally, we looked forward to the development trends of HCFGLs. As an emerging type of fiber laser, HCFGLs have unique advantages in wavelength extension and achieving narrow linewidth lasers, and they are poised to play a more significant role in the future. Currently, this new type of laser is still in its infancy and faces several challenges, including the need to increase output power, realize abundant output spectra, and improve compactness. With the deepening of research, we believe that these issues will be effectively addressed, paving the way for the practical application of these lasers in various fields.

## Data Availability

The data underlying the results presented in this paper are not publicly available at this time but may be obtained from the authors upon reasonable request.
